# Altered Metabolic Profiles of the Plasma of Patients with Amyotrophic Lateral Sclerosis

**DOI:** 10.3390/biomedicines9121944

**Published:** 2021-12-18

**Authors:** Kuo-Hsuan Chang, Chia-Ni Lin, Chiung-Mei Chen, Rong-Kuo Lyu, Chun-Che Chu, Ming-Feng Liao, Chin-Chang Huang, Hong-Shiu Chang, Long-Sun Ro, Hung-Chou Kuo

**Affiliations:** 1 Department of Neurology, Chang Gung Memorial Hospital Linkou Medical Center and College of Medicine, Chang Gung University, Taoyuan 333, Taiwan; gophy5128@cgmh.org.tw (K.-H.C.); cmchen@adm.cgmh.org.tw (C.-M.C.); lyu5172@adm.cgmh.org.tw (R.-K.L.); 1939chu@adm.cgmh.org.tw (C.-C.C.); mingfengliao@adm.cgmh.org.tw (M.-F.L.); cch0537@adm.cgmh.org.tw (C.-C.H.); hschang@cgmh.org.tw (H.-S.C.); cgrols@adm.cgmh.org.tw (L.-S.R.); 2 Department of Laboratory Medicine, Chang Gung Memorial Hospital, Taoyuan 333, Taiwan; chianilin@cgmh.org.tw; 3 Department of Medical Biotechnology and Laboratory Science, College of Medicine, Chang Gung University, Taoyuan 333, Taiwan

**Keywords:** amyotrophic lateral sclerosis, biomarker, metabolomics, creatinine, asymmetric dimethylarginine, methionine, phosphatidylcholine, sphingomyelin

## Abstract

Currently, there is no objective biomarker to indicate disease progression and monitor therapeutic effects for amyotrophic lateral sclerosis (ALS). This study aimed to identify plasma biomarkers for ALS using a targeted metabolomics approach. Plasma levels of 185 metabolites in 36 ALS patients and 36 age- and sex-matched normal controls (NCs) were quantified using an assay combining liquid chromatography with tandem mass spectrometry and direct flow injection. Identified candidates were correlated with the scores of the revised ALS Functional Rating Scale (ALSFRS-r). Support vector machine (SVM) learning applied to selected metabolites was used to differentiate ALS and NC subjects. Forty-four metabolites differed significantly between ALS and NC subjects. Significant correlations with ALSFRS-r score were seen in 23 metabolites. Six of them showing potential to distinguish ALS from NC—asymmetric dimethylarginine (area under the curve (AUC): 0.829), creatinine (AUC: 0.803), methionine (AUC: 0.767), PC-acyl-alkyl C34:2 (AUC: 0.808), C34:2 (AUC: 0.763), and PC-acyl-acyl C42:2 (AUC: 0.751)—were selected for machine learning. The SVM algorithm using selected metabolites achieved good performance, with an AUC of 0.945. In conclusion, our findings indicate that a panel of metabolites were correlated with disease severity of ALS, which could be potential biomarkers for monitoring ALS progression and therapeutic effects.

## 1. Introduction

Amyotrophic lateral sclerosis (ALS) is a progressive neurodegenerative disease caused by the progressive degeneration of motor neurons [1]. The pathogenesis of neurodegeneration in ALS has not been fully disclosed. Several pathogenic pathways have been identified, including abnormal proteostasis; mitochondrial, autophagic, and metabolic dysfunction; increased oxidative stress and inflammation; and failure of axonal transport [2,3]. Approximately 10% of patients with ALS have a family history of the disease, with the remainder of cases classified as sporadic [4]. Currently, effective treatments to prevent disease progression or modify the disease course for ALS are few. Riluzole prolongs survival by 2–3 months [5], whereas edaravone mildly improves patient mobility [6]. The main hurdle in developing an effective treatment for ALS is the lack of objective and useful biomarkers to indicate early disease progression and to test the efficacy of potential treatments. Establishing ALS-specific molecular biomarkers, particularly in blood, could help in revealing pathogenesis, detecting the disease at preclinical or early stages, indicating disease progression, and monitoring the effect of therapeutic measures by potential disease modifiers.

Metabolites are compartment-specific in the sense that they may participate in different biochemical reactions according to whether they are found in bodily fluids, cells, or tissues. The profiles of metabolites are produced by catabolic and anabolic processes in different tissues, serving to reflect dysregulated cellular and metabolic processes. In ALS, the metabolic profiles revealed by various metabolomics platforms show a disturbance of a number of ALS-associated metabolic pathways, including amino acid, pyruvate, and lipid metabolism [7,8,9,10]. Altered levels of sphingolipids have been reported in the plasma of ALS patients [11,12,13]. Plasma levels of arginine, proline, lysine, histidine, and polyamines are also changed in ALS patients [7,12,14,15,16]. Herein, we measured plasma levels of 185 metabolites in ALS patients to identify candidate metabolic biomarker(s) and pathomechanistic pathway(s) of ALS by using an assay combining liquid chromatography–tandem mass spectrometry (LC–MS/MS) and direct flow injection. Biomarker candidates that were correlated with clinical parameters were subjected to machine learning to establish an algorithm for diagnosing ALS.

## 2. Materials and Methods

### 2.1. Standard Protocol Approvals, Registrations, and Patient Consents

The study protocol was approved by the Institutional Review Boards of Chang Gung Memorial Hospital (ethical licenses No: 201601762B0, 13 January 2017; 201601762B0C501, 26 October 2018; 201601762B0C502, 17 February 2020; 201601762B0C601, 25 March 2020). Written informed consent was obtained from all recruited patients and controls. Patients with ALS were recruited from the neurology clinics of Chang Gung Memorial Hospital between December 2018 and November 2020. The diagnosis of ALS was based on the Awaji criteria for diagnosis of ALS [17]. Demographic information, laboratory data, scores on the revised ALS Functional Rating Scale (ALSFRS-r) [18], and medications were recorded for each patient. Sex- and age-matched normal controls (NCs) were randomly recruited from neurology outpatient clinics. The number of hexanucleotide repeat expansions within the *C9ORF72* gene was recorded for patients with a family history of ALS. All subjects were free from systemic infection, chronic renal failure, cardiac or liver dysfunction, malignancies, autoimmune diseases, stroke, or any neurodegenerative diseases other than ALS. Blood samples for metabolomics analysis were collected from subjects who were asked to fast overnight for 12 h before collection.

### 2.2. Determining Concentrations of Plasma Metabolites

Plasma samples from 36 patients with ALS and 36 NCs were collected to quantify 185 metabolites belonging to acylcarnitines, amino acids, biogenic amines, glycerophospholipids, sphingomyelins (SMs), and sugars, using the targeted Absolute IDQ^®^ p180 kit (Biocrates Life Sciences AG, Innsbruck, Austria). Plasma samples were centrifuged at 13,000× *g*. The supernatant (10 μL) was loaded onto a filter paper, dried under a nitrogen flow, and derivatized using 5% phenyl isothiocyanate (20 μL) for 20 min. Ammonium acetate (5 nM, 300 μL) in methanol was added to the sample spots on the filter paper after they were dried under a nitrogen flow for 45 min. The extracts were then injected onto an Acquity UPLC BEH C18 (2.1 mm × 75 mm, 1.7 μm particle size; Waters Corp., Milford, MA, USA) at 50 °C to separate amino acids and biogenic amines into negative electrospray ionization and multiple reaction monitoring (MRM) mode. Then, sphingolipids, sugars, acylcarnitines, and glycerophospholipids were separated by flow injection analysis–tandem mass spectrometry (FIA–MS/MS). TargetLynxTM (Waters Corp.) with an external 7-point calibration was used to quantify LC data. The converted flow injection analysis data were imported into the Biocrates^®^ MetIDQ™ software (Biocrates Life Sciences AG).

### 2.3. Statistical Analysis

Continuous variables were presented as mean and standard deviation, and analyzed by Student’s *t*-test or analysis of covariance (ANCOVA) with false discovery rate (FDR) adjustment to correct multiple tests, where appropriate. Categorical variables were presented as counts and percentages and analyzed using the chi-squared test. The clinical variables and metabolites were analyzed with orthogonal partial-least-squares-discriminant-analysis (OPLS-DA) using the web-based metabolomics software MetaboAnalyst 5.0 (McGill University, Montreal, QC, Canada). The variable importance in the projection (VIP) of each metabolite in the model was calculated to indicate its contribution to the classification. A higher VIP value indicates a stronger contribution to the discrimination between groups. VIP values greater than 1.0 were considered significantly different. Pearson correlations were applied to evaluate the relationship between the levels of metabolites and clinical parameters. An analysis of the receiver operating characteristic (ROC) curve was used to measure the ability of individual molecules to distinguish ALS patients from NCs. Selected molecules were further introduced to the support vector machine (SVM) algorithm. The performance estimation of the models was further analyzed by ROC curves generated by Monte-Carlo cross-validation using balanced subsampling. Two-thirds of the subjects were used to build classification models, which were then validated using the remaining third. To produce a smooth ROC curve, 100-fold cross-validations were performed, and the results were averaged to generate the plot.

### 2.4. Data Availability

The datasets generated during the current study are available from the corresponding author on reasonable request.

## 3. Results

### 3.1. Participants

A total of 36 patients with ALS and 36 sex- and age-matched NCs were recruited into this study (Table 1). A family history of ALS was noted in one male patient, who also carried hexanucleotide repeat expansions within the *C9ORF72* gene. Patients with ALS had a significantly lower body mass index (BMI, 21.36 ± 4.52) than NCs (25.22 ± 3.64, *p* < 0.001). The levels of preprandial glucose, triglyceride, cholesterol, high-density lipoprotein, and low-density lipoprotein were similar between the ALS and NC groups. The interval between symptom onset and blood draw was 2.89 ± 3.49 years. The ALSFRS-r scores of the ALS patients were 27.14 ± 13.93. Eighty patients (50%) with ALS were treated with riluzole. None of them were treated with edaravone, given that edaravone remained unavailable in Taiwan. The spinal-onset subtype was seen in 33 patients (91.67%), followed by bulbar-onset (5.56%) and respiratory-onset (2.78%) subtypes.

### 3.2. Targeted Metabolomics Analysis

**Figure 1:**
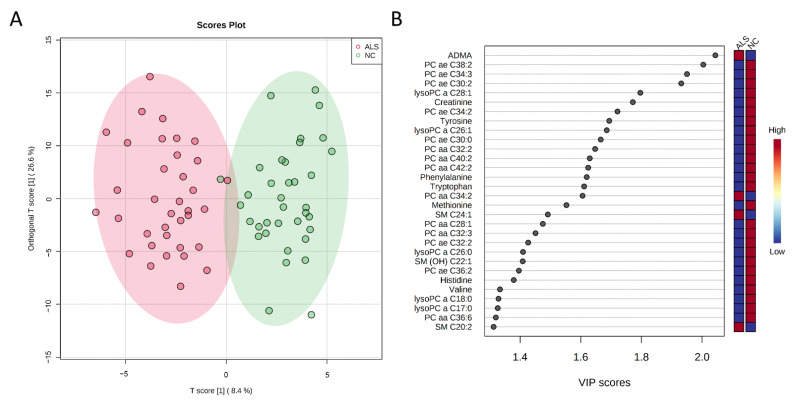
Orthogonal partial least squares-discriminant analysis (OPLS-DA) between normal controls (NCs, *n* = 36) and patients with amyotrophic lateral sclerosis (ALS) (*n* = 36). (**A**) OPLS-DA shows a separation of metabolites between two groups (R^2^Y = 0.389, Q^2^ = 0.311). R^2^Y, cumulative variation in the Y matrix; Q^2^, predictive performance of the model. (**B**) The top 30 metabolites with variable importance in the projection (VIP) score > 1.0 indicating their contribution to the classification in the OPLS-DA model. aa: acyl-acyl; ae: acyl-alkyl; ADMA: asymmetric dimethylarginine; OH: hydroxy; lysoPC: lysophosphatidylcholine; PC: phosphatidylcholine; SM: sphingomyelin.

Among 185 metabolites studied using the AbsoluteIDQ^®^ p180 Kit, 131 were detectable among all subjects. The OPLS-DA for all metabolites could separate ALS from NCs (R2Y, 0.389; Q2, 0.311, Figure 1A), while 53 metabolites had a VIP score > 1.0 (Figure 1B and Supplementary Table S1). After adjustment for BMI, 44 metabolites had significantly different plasma levels between ALS and NCs, including 31 phosphatidylcholines (PCs), 4 SMs, and 9 biogenic amines/amino acids (Table 2 and Supplementary Table S2). The 43 metabolites with VIP score > 1.0 and altered plasma levels in ALS patients were selected as biomarker candidates for further correlation with clinical parameters in the ALS group.

### 3.3. Clustering and Pathway Enrichment Analysis

The hierarchical clustering heatmaps using the selected biomarker candidates and the clinical parameters of ALS patients are shown in Supplementary Figure S1A. Most of the patients with ALS were aggregated in the same cluster. Metabolomics pathway enrichment analysis identified 17 over-represented pathways among the differential metabolites (Supplementary Figure S1B and Supplementary Table S3). The leading pathways associated with ALS were “phenylalanine, tyrosine, and tryptophan biosynthesis,” “tyrosine metabolism,” and “ubiquinone and other terpenoid-quinone biosynthesis.”

### 3.4. Correlation Analysis between Metabolites and Disease Severity

The levels of these metabolites were further correlated with ALSFRS-r scores (Table 3). Positive correlations with ALSFRS-r scores were seen in plasma levels of 22 metabolites, particularly creatinine (*r* = 0.61, *p* < 0.001), PC acyl-alkyl (ae) C34:3 (*r* = 0.55, *p* < 0.001), and SM hydroxy (OH) C22:1 (*r* = 0.53, *p* = 0.001). Plasma levels of asymmetric dimethylarginine (ADMA) were negative correlated with ALSFRS-r scores (*r* = −0.41, *p* = 0.012). These 22 metabolites were selected for further ROC curve analysis.

### 3.5. Classification Models for Differentiating Patients with Amyotrophic Lateral Sclerosis and Controls

**Figure 2:**
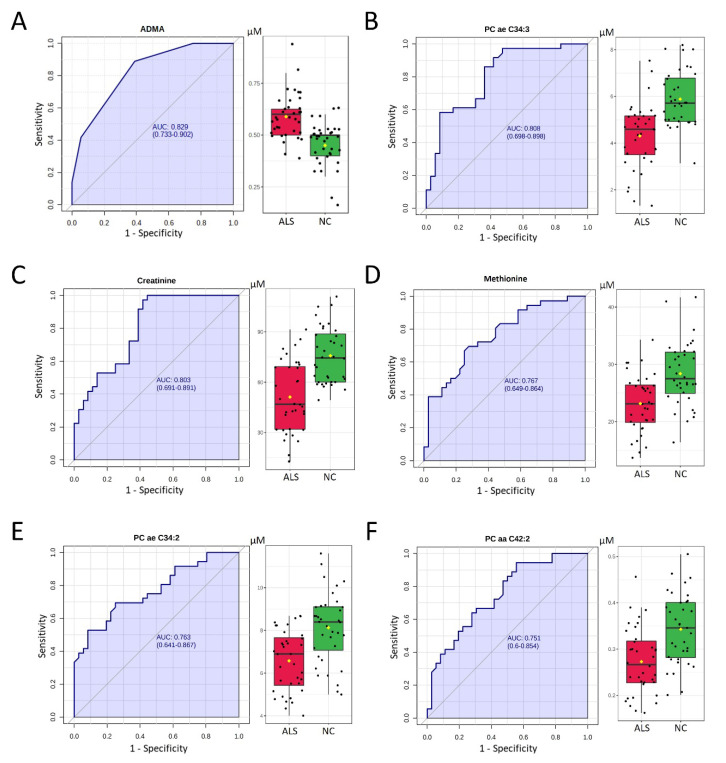
Receiver operating characteristic (ROC) curve (left panel) and box plot analysis (right panel) of plasma levels of (**A**) asymmetric dimethylarginine (ADMA), (**B**) phosphatidylcholine (PC) acyl-alkyl (ae) C34:3, (**C**) creatinine, (**D**) methionine, (**E**) PC ae C34:2, and (**F**) PC acyl-acyl (aa) C42:2 for the diagnosis of amyotrophic lateral sclerosis (ALS). The area under the ROC curve (AUC) is in shadow. The black center line in the box plot denotes the median, while the blue or green boxes contain the 25^th^ to 75^th^ percentiles for patients with ALS or NC, respectively. The black whiskers mark the 5^th^ and 95^th^ percentiles, and mean values are marked with yellow dots. Two-tailed Student’s *t*-test. NC: age- and sex-matched controls.

**Figure 3:**
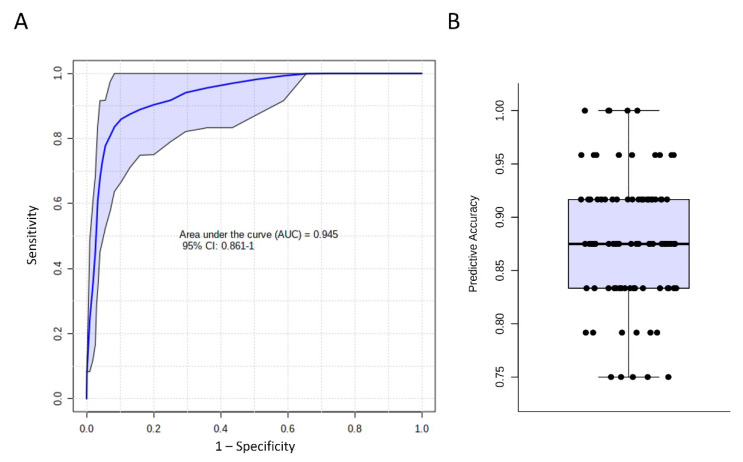
Diagnosis of amyotrophic lateral sclerosis by identified metabolites. (**A**) Receiver operating characteristic analysis using asymmetric dimethylarginine (ADMA), phosphatidylcholine (PC) acyl-alkyl (ae) C34:3, creatinine, methionine, PC ae C34:2, and PC acyl-acyl (aa) C42:2 by support vector machine. One-hundred-fold cross-validations were performed, and the results were averaged to generate the plot. The 95% confidence intervals are indicated as the blue shaded area. (**B**) Predictive accuracy of cross-validations. The average accuracy was 0.88. CI: Confidence Interval.

Among 22 selected metabolites, six demonstrated an adequate potential to distinguish ALS and NC, with an area under the ROC curve (AUC) greater than 0.750. ADMA showed the greatest AUC (0.829) to distinguish ALS and NC, followed by PC ae C34:3 (AUC: 0.808), creatinine (AUC: 0.803), methionine (AUC: 0.767), PC ae C34:2 (AUC: 0.763), and PC acyl-acyl (aa) C42:2 (AUC: 0.751, Figure 2). The SVM algorithm using these six metabolites demonstrated a good ability to separate ALS from NC (AUC: 0.945, Figure 3). The average accuracy for 100 cross-validations was 0.88. These results support the potential of using a combination of identified metabolite biomarkers to establish a machine learning algorithm for ALS diagnosis.

## 4. Discussion

**Figure 4:**
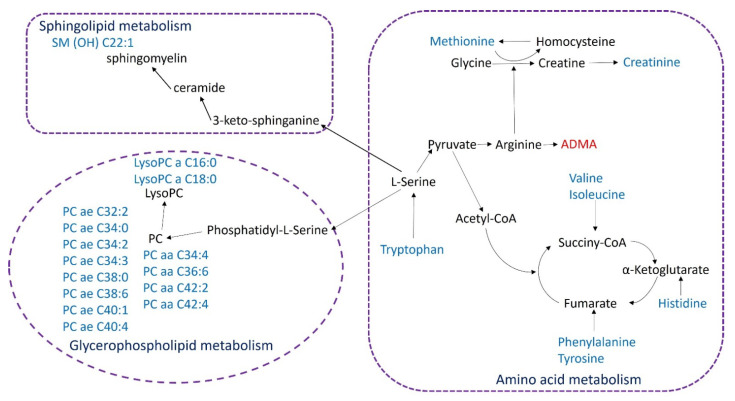
Network of the identified key biomarkers and pathways in plasma of patients with amyotrophic lateral sclerosis. The metabolites colored blue or red represent declining or increasing levels, respectively. Aa: acyl-acyl; ae: acyl-alkyl; ADMA: asymmetric dimethylarginine; OH: hydroxy; lysoPC: lysophosphatidylcholine; PC: phosphatidylcholine; SM: sphingomyelin.

By extensively examining the plasma levels of 185 metabolites in ALS patients, we found profound metabolomic alterations in 31 PCs, 4 SMs, and 9 amino acids or amines. These metabolites were involved in the biosynthesis or metabolism of sphingolipids, glycerophospholipids, and amino acids, including tyrosine, tryptophan, methionine, histidine, valine, and isoleucine (Figure 4). Twenty-two metabolites were demonstrated to correlate with ALSFRS-r scores. Plasma levels of six metabolites, including ADMA, creatinine, methionine, PC aa C42:2, PC ae C34:3, and C34:2, demonstrated the potential to distinguish ALS patients from NCs. Using these metabolites, the SVM machine learning algorithm demonstrated a good ability to separate ALS from NCs. In addition to identifying significant alterations in lipid and amino acid metabolism, these results also support the role of plasma metabolomic analysis to detect ALS and monitor disease progression.

PCs are abundant lipoproteins of cell membranes and play critical roles in membrane structure and cellular signal transduction [19]. Inhibition of PC synthesis can trigger apoptosis [20]. Increased levels of PC C36:4 have been observed in the cerebrospinal fluid (CSF) of ALS patients [9]. By contrast, decreased levels of total PCs have been reported in the plasma of ALS patients and have shown significant increases in follow-up samples of patients [21]. Our study further elucidated the reduced plasma levels in ALS patients of 31 specific PCs, 14 of which were significantly correlated with the ALSFRS-r scores. Reductions in PCs in plasma are also seen in other neurodegenerative diseases, such as Alzheimer’s (AD) and Huntington’s disease (HD) [22,23]. The specifics of these lipid markers need to be confirmed by further investigation.

SMs, which contain acyl chains that vary in length from long-chain to very-long-chain fatty acids [24], are indispensable sphingolipids in mammalian cell membranes [25]. The role of SMs in ALS pathogenesis remains elusive. Increased levels of SMs have been reported in the spinal cords of superoxide dismutase (SOD)-overexpressing mice and ALS patients [26]. Elevated levels of SMs are present in both the grey matter and ventral white matter tissue samples of ALS patients [27]. On the other hand, the levels of SM in the spinal cord of *SOD1*-G86R mice were significantly down-regulated [28]. In the plasma of ALS patients, we found elevated levels of SM C24:1 and SM C20:2, but decreased levels of SM (OH) C22:1 and SM (OH) C24:1. The hydroxylation of SMs may be important in maintaining growth and mediating the fatty acid composition of sphingolipids [29,30]. Further studies are warranted to confirm the role of SMs and their hydroxylation in the pathogenesis of ALS.

Derived from the high-energy product creatine, blood creatinine is widely used as a biomarker of kidney function. Previous studies have demonstrated decreased creatinine levels in those with motor neuron diseases, including ALS [10,12,31], spinal muscular atrophy (SMA) [32], and spinal and bulbar muscular atrophy (SBMA) [31]. Consistent with our study, lower levels of creatinine have been observed in the plasma [10,12,31], serum [32], and CSF [14] of ALS patients. Lawton et al. [12] also demonstrated a correlation between plasma levels of creatinine and the scores of ALSFRS-r. Decreased creatinine levels were also observed in the serum of SMA patients, which correlated with increasing disease severity [32]. Serum creatinine levels decrease before the onset of clinical symptoms in SBMA patients [31]. Creatine, synthesized primarily in the liver from the methylation of guanidinoacetic acid by guanidinoacetate N-methyltransferase, is transported through the blood to muscles [33]. Creatine is nonenzymatically converted to creatinine in muscles, which is excreted by the kidneys into the urine [33]. Therefore, the reduction in creatinine in plasma could be a biomarker reflecting the loss of skeletal muscle in patients with ALS. On the other hand, creatine also plays an important role in brain energetics [34]. Future studies in patients with genetically defined ALS will be warranted to clarify the role of creatinine in the prodromal stage of ALS.

Valine and isoleucine are branched-chain essential amino acids (BCAAs). The catabolism of BCAA majorly takes place in the skeletal muscle by branched-chain amino-acid aminotransferase to form glutamate and the corresponding branched-chain keto acids [35]. Reduced blood levels of BCAAs have been discovered in those with neurodegenerative diseases, including AD and HD. Serum levels of valine are reduced in patients with AD [36]. Low levels of BCAAs have also been identified in the plasma of HD patients [22,37]. Our study similarly detected low plasma levels of valine and isoleucine in ALS patients. By contrast, Wuolikainen et al. [14] detected high plasma levels of BCAAs in patients with ALS. It is also worth noting that the supplementary intake of BCAAs failed to demonstrate a clinical benefit in ALS patients [14], suggesting that these metabolites are not appropriate targets for treating ALS.

Phenylalanine, tyrosine, and tryptophan are essential components for the production of several neurotransmitters, including epinephrine, norepinephrine, dopamine, and serotonin [38,39]. As a precursor of histamine, the transportation of histidine across the blood–brain barrier is essential for maintaining the histaminergic nervous system, involved in sleeping, eating, and mood stability [40]. Methionine is required for providing a methyl group for the synthesis of creatine and PCs, as well as for DNA methylation [41]. Our study found that the levels of phenylalanine, methionine, tryptophan, and histidine were reduced and positively correlated with the ALSFRS-r scores in ALS patients. Similarly, Ilzecka et al. [42] found reduced plasma levels of tyrosine in patients with ALS. ALS patients also had lower CSF levels of tryptophan [43]. By contrast, CSF levels of tyrosine, histidine, and phenylalanine were increased in ALS patients [15,43]. The role of these amino acids in ALS pathogenesis requires further elucidation.

ADMA, an endogenous inhibitor of endothelial nitric oxide synthase [44], plays a role in mediating endothelial dysfunction and the risk of cardiovascular disease [45]. In the CSF of patients with ALS, the ADMA concentration has been reported to be significantly decreased [46]. Our study showed elevated ADMA in the plasma of ALS patients. The plasma levels of ADMA were also negatively correlated with the ALSFRS-r scores.

Goutman et al. [10] applied SVM to 259 metabolites with an AUC of 0.96 to differentiate ALS patients from NCs. Using six selected metabolites, our model demonstrated comparable performance, with an AUC of 0.945, providing a potentially easily accessible tool for ALS diagnosis. Creatinine, methionine, and ADMA were used by both machine-learning algorithms, reinforcing the participation of these molecules in ALS pathogenesis. However, the relatively small number of subjects raises concerns about overfitting and inadequate generalization. The sensitivity to differentiate ALS patients from those with other neurodegenerative diseases was uncertain. To validate our results, future metabolomics studies should recruit a larger number of patients with ALS and other neurodegenerative diseases.

Genetic factors can cause differences in metabolites and should be considered a contributory mechanism to the manifestation of ALS. For example, mutations in the *SOD1* and *C9ORF72* genes are found in the majority of those with familial ALS [47]. We found hexanucleotide repeat expansions within the *C9ORF72* gene in a patient, but we did not perform a *SOD1* genetic study.

There are limitations to this study. Currently, molecular diagnosis of ALS remains unavailable. The power in our study may not have been sufficient to detect smaller changes in metabolites associated with ALS. Some unknown interactions of medications or other factors may also have partially contributed to the metabolic differences between groups. Our results need to be validated in a different cohort. The temporal changes in these metabolites should be studied using a longitudinal follow-up of a larger cohort of ALS patients. Further investigations are warranted to determine whether metabolomics biomarkers identified are related to the primary process of motor neuron degeneration in ALS and whether certain biomarkers are more closely aligned with specific ALS subtypes. Nevertheless, our study clearly captures important features of the metabolomics of the plasma of ALS patients (Figure 4). These metabolic changes provide potential avenues for investigating pathogenesis, monitoring clinical progression, and assessing treatment efficacy in ALS patients. The metabolomics-based machine learning algorithm also showed high potential to assist in the diagnosis of ALS.

## 5. Conclusions

Using targeted metabolomics, we identified several metabolites significantly correlated with disease severity in patients with ALS. Although the sample size was limited by the rarity of ALS, we found key metabolites that are probably involved in the pathogenesis of ALS. These findings also provide a panel of biomarkers for ALS diagnosis and progression, as well for potential new therapeutic targets for ALS.

## Figures and Tables

**Table 1:** Demographic characteristics and blood biochemical parameters of included patients with amyotrophic lateral sclerosis (ALS) and age- and sex-matched normal controls (NCs).

	NC (*n* = 36)	ALS (*n* = 36)
Age (years)	57.89 ± 6.51	57.31 ± 9.86
Male (%)	21 (0.58%)	21 (0.58%)
BMI *	25.22 ± 3.64	21.36 ± 4.52
Triglyceride (mg/dL)	131.33 ± 78.54	107.16 ± 49.21
Cholesterol (mg/dL)	198.42 ± 65.19	184.99 ± 52.96
HDL (mg/dL)	48.48 ± 17.45	54.55 ± 16.44
LDL (mg/dL)	121.16 ± 49.52	115.77 ± 36.65
Pre-prandial glucose (mg/dL)	106.14 ± 46.05	95.89 ± 11.48
Glycohemoglobin (%)	6.04 ± 2.00	5.67 ± 0.64
Diabetes (%)	4 (11.11%)	5 (13.89%)
Time between symptom onset and blood draw (years)		2.89 ± 3.49
Family history of ALS (%)		1 (2.78%)
Riluzole (%)		18 (50%)
ALSFRS-r		27.14 ± 13.93
Onset subtype		
Spinal		33 (91.67%)
Bulbar		2 (5.56%)
Respiratory		1 (2.78%)

ALSFRS-r: revised Amyotrophic Lateral Sclerosis Functional Rating Scale; BMI: body mass index; HDL: high-density lipoprotein; LDL: low-density lipoprotein. *: Significant difference between NC and ALS. *p* < 0.05. Two-tailed Student’s *t*-test.

**Table 2:** Top 15 plasma metabolites that significantly differ between patients with amyotrophic lateral sclerosis (ALS) and age- and sex-matched normal controls (NCs).

Compound Name	NC (*n* =36)	ALS (*n* =36)	*p* Value
ADMA (μM)	0.45 ± 0.10	0.59 ± 0.11	*p* < 0.001
Creatinine (μM)	75.81 ± 16.78	51.16 ± 20.91	*p* < 0.001
PC ae C34:3 (μM)	5.88 ± 1.19	4.31 ± 1.41	*p* < 0.001
PC ae C38:2 (μM)	1.43 ± 0.68	0.81 ± 0.41	*p* < 0.001
PC ae C30:2 (μM)	0.06 ± 0.01	0.05 ± 0.01	*p* < 0.001
Tyrosine (μM)	75.29 ± 15.68	59.58 ± 13.91	*p* < 0.001
PC ae C34:2 (μM)	8.13 ± 1.65	6.57 ± 1.41	*p* < 0.001
Tryptophan (μM)	65.06 ± 14.17	52.35 ± 10.85	*p* < 0.001
Methionine (μM)	28.38 ± 5.57	23.16 ± 5.03	*p* = 0.001
Phenylalanine (μM)	71.29 ± 9.95	61.02 ± 11.23	*p* = 0.001
PC aa C42:2 (μM)	0.34 ± 0.07	0.27 ± 0.07	*p* = 0.001
PC ae C30:0 (μM)	0.20 ± 0.06	0.15 ± 0.04	*p* = 0.001
PC aa C40:2 (μM)	0.41 ± 0.13	0.31 ± 0.08	*p* = 0.002
PC aa C34:2 (μM)	171.00 ± 20.29	190.19 ± 21.92	*p* = 0.002
lysoPC a C26:1 (μM)	0.05 ± 0.02	0.04 ± 0.01	*p* = 0.002

*p* value: analysis of covariance (ANCOVA) adjustment for body mass index, with false discovery rate correction. aa: acyl-acyl; ae: acyl-alkyl; ADMA: asymmetric dimethylarginine; OH: hydroxy; lysoPC: lysophosphatidylcholine; PC: phosphatidylcholine; SM: sphingomyelin.

**Table 3:** The correlations between plasma levels of identified metabolites and scores of the revised Amyotrophic Lateral Sclerosis rating scale (ALSRS-r).

Compound Name	ALSRS-r	*p* Value
Creatinine	0.61	<0.001
PC ae C34:3	0.55	<0.001
SM (OH) C22:1	0.53	0.001
PC ae C38:6	0.52	0.001
Methionine	0.47	0.004
PC aa C42:4	0.43	0.009
PC aa C42:2	0.43	0.009
PC ae C40:1	0.42	0.011
ADMA	−0.42	0.012
Tryptophan	0.41	0.012
PC ae C38:0	0.40	0.016
Valine	0.40	0.016
Phenylalanine	0.39	0.018
Histidine	0.38	0.023
PC ae C34:0	0.37	0.024
lysoPC a C18:0	0.37	0.029
PC aa C36:6	0.34	0.040
PC ae C32:2	0.34	0.040
lysoPC a C16:0	0.34	0.043
PC aa C34:4	0.33	0.047
PC ae C34:2	0.33	0.048
PC ae C40:4	0.33	0.049

aa: acyl-acyl; ae: acyl-alkyl; ADMA: asymmetric dimethylarginine; OH: hydroxy; lysoPC: lysophosphatidylcholine; PC: phosphatidylcholine; SM: sphingomyelin.

## Data Availability

The datasets generated during the current study are available from the corresponding author on reasonable request.

## Supplementary Materials

The following are available online at https://www.mdpi.com/article/10.3390/biomedicines9121944/s1, Figure S1: The correlation between selected metabolites and clinical parameters, Table S1: Metabolites with VIP score > 1, Table S2: Plasma metabolites that significantly differ between patients with amyotrophic lateral sclerosis (ALS) and age- and sex-matched normal controls (NCs), Table S3: The pathway analysis of metabolites with altered levels in the plasma of patients with amyotrophic lateral sclerosis.
