# Supplementary material for: Altered Metabolic Profiles of the Plasma of Patients with Amyotrophic Lateral Sclerosis
Source: Biomedicines. 2021 Dec 18;9(12):1944. doi: 10.3390/biomedicines9121944 (PMC8699018; doi:10.3390/biomedicines9121944)

SUPPLEMENTAL MATERIAL

Supplementary Table S1. Metabolites with VIP score > 1.

Metabolite	VIP score
ADMA	2.044
PC ae C38:2	2.004
PC ae C34:3	1.950
PC ae C30:2	1.932
lysoPC a C28:1	1.796
Creatinine	1.772
PC ae C34:2	1.721
Tyrosine	1.694
lysoPC a C26:1	1.686
PC ae C30:0	1.666
PC aa C32:2	1.647
PC aa C40:2	1.630
PC aa C42:2	1.624
Phenylalanine	1.620
Tryptophan	1.611
PC aa C34:2	1.606
Methionine	1.553
SM C24:1	1.491
PC aa C28:1	1.475
PC aa C32:3	1.451
PC ae C32:2	1.427
lysoPC a C26:0	1.410
SM (OH) C22:1	1.409
PC ae C36:2	1.396
Histidine	1.379
Valine	1.333
lysoPC a C18:0	1.329
lysoPC a C17:0	1.326
PC aa C36:6	1.320
SM C20:2	1.313
lysoPC a C28:0	1.256
PC ae C38:6	1.236
lysoPC a C16:0	1.226

PC ae C40:2	1.221
PC ae C38:3	1.216
PC ae C44:5	1.210
PC ae C40:3	1.208
PC ae C38:0	1.204
PC aa C34:4	1.159
PC ae C34:0	1.150
PC aa C42:4	1.148
SM (OH) C24:1	1.135
PC ae C36:3	1.127
PC aa C42:6	1.118
PC ae C40:1	1.112
Citrate	1.106
PC aa C30:0	1.085
PC ae C40:4	1.077
Isoleucine	1.062
PC ae C36:1	1.059
PC aa C36:1	1.043
lysoPC a C14:0	1.042
SM C18:1	1.008

aa: acyl-acyl; ae: acyl-alkyl; ADMA: asymmetric dimethylarginine; OH: hydroxy; lysoPC: lysophosphatidylcholine; PC: phosphatidylchololine; SM: sphinogomyelin; VIP: variable importance.

Supplementary Table S2. Plasma metabolites which significantly differ between patients with amyotrophic lateral sclerosis (ALS) and age- and sex-matched normal controls (NC).

Compound name	NC (n =36)	ALS (n =36)	P value
ADMA (μM)	0.45 ± 0.10	0.59 ± 0.11	$P < 0.001$
Creatinine (μM)	75.81 ± 16.78	51.16 ± 20.91	$P < 0.001$
PC ae C34:3 (μM)	5.88 ± 1.19	4.31 ± 1.41	$P < 0.001$
PC ae C38:2 (μM)	1.43 ± 0.68	0.81 ± 0.41	$P < 0.001$
PC ae C30:2 (μM)	0.06 ± 0.01	0.05 ± 0.01	$P < 0.001$
Tyrosine (μM)	75.29 ± 15.68	59.58 ± 13.91	$P < 0.001$
PC ae C34:2 (μM)	8.13 ± 1.65	6.57 ± 1.41	$P < 0.001$
Tryptophan (μM)	65.06 ± 14.17	52.35 ± 10.85	$P < 0.001$
Methionine (μM)	28.38 ± 5.57	23.16 ± 5.03	$P = 0.001$
Phenylalanine (μM)	71.29 ± 9.95	61.02 ± 11.23	$P = 0.001$
PC aa C42:2 (μM)	0.34 ± 0.07	0.27 ± 0.07	$P = 0.001$
PC ae C30:0 (μM)	0.20 ± 0.06	0.15 ± 0.04	$P = 0.001$
PC aa C40:2 (μM)	0.41 ± 0.13	0.31 ± 0.08	$P = 0.002$
PC aa C34:2 (μM)	171.00 ± 20.29	190.19 ± 21.92	$P = 0.002$
lysoPC a C26:1 (μM)	0.05 ± 0.02	0.04 ± 0.01	$P = 0.002$
lysoPC a C28:1 (μM)	0.16 ± 0.04	0.13 ± 0.03	$P = 0.003$
Histidine (μM)	89.37 ± 9.24	80.90 ± 10.49	$P = 0.004$
SM C24:1 (μM)	77.50 ± 13.47	92.81 ± 21.98	$P = 0.005$
Valine (μM)	280.19 ± 54.71	236.08 ± 52.43	$P = 0.006$
PC aa C32:2 (μM)	2.25 ± 0.74	1.60 ± 0.90	$P = 0.009$
PC aa C28:1 (μM)	1.88 ± 0.47	1.53 ± 0.44	$P = 0.009$
SM (OH) C22:1 (μM)	19.36 ± 3.54	16.17 ± 4.57	$P = 0.009$
PC ae C32:2 (μM)	0.43 ± 0.10	0.35 ± 0.10	$P = 0.010$
PC ae C38:6 (μM)	4.65 ± 1.26	3.69 ± 1.28	$P = 0.010$
PC aa C32:3 (μM)	0.23 ± 0.05	0.20 ± 0.04	$P = 0.010$
lysoPC a C18:0 (μM)	34.26 ± 8.10	28.38 ± 7.95	$P = 0.014$
SM C20:2 (μM)	0.50 ± 0.12	0.60 ± 0.16	$P = 0.016$
lysoPC a C16:0 (μM)	111.80 ± 21.97	96.75 ± 20.17	$P = 0.016$
PC aa C36:6 (μM)	0.38 ± 0.14	0.28 ± 0.15	$P = 0.019$
lysoPC a C17:0 (μM)	1.79 ± 0.49	1.48 ± 0.41	$P = 0.019$
lysoPC a C26:0 (μM)	0.15 ± 0.05	0.11 ± 0.03	$P = 0.019$

PC ae C40:2 (μM)	1.50 ± 0.36	1.27 ± 0.30	*P* = 0.019
PC ae C38:0 (μM)	1.05 ± 0.31	0.86 ± 0.26	*P* = 0.026
PC ae C44:5 (μM)	0.99 ± 0.23	1.18 ± 0.34	*P* = 0.028
PC ae C36:2 (μM)	9.55 ± 2.28	8.24 ± 1.76	*P* = 0.030
PC aa C42:4 (μM)	0.21 ± 0.06	0.18 ± 0.04	*P* = 0.032
PC aa C42:6 (μM)	0.35 ± 0.09	0.30 ± 0.08	*P* = 0.034
PC ae C40:1 (μM)	1.24 ± 0.32	1.04 ± 0.32	*P* = 0.037
SM (OH) C24:1 (μM)	1.83 ± 0.04	1.59 ± 0.38	*P* = 0.038
PC aa C42:5 (μM)	0.32 ± 0.09	0.27 ± 0.06	*P* = 0.038
Isoleucine (μM)	81.73 ± 19.33	70.73 ± 16.77	*P* = 0.038
PC ae C34:0 (μM)	0.73 ± 0.22	0.61 ± 0.18	*P* = 0.039
PC aa C34:4 (μM)	0.68 ± 0.21	0.55 ± 0.21	*P* = 0.039
PC ae C40:4 (μM)	1.62 ± 0.31	1.44 ± 0.32	*P* = 0.043

P value: analysis of covariance (ANCOVA) adjustment for body mass index, with false discovery rate correction.

aa: acyl-acyl; ae: acyl-alkyl; ADMA: asymmetric dimethylarginine; OH: hydroxy; lysoPC: lysophosphatidylcholine; PC: phosphatidylchololine; SM: sphinogomyelin.

Supplementary Table S3. Pathway analysis of metabolites with altered levels in the plasma of patients with amyotrophic lateral sclerosis.

Pathway Name	Match status (matched/total)	*P* value	Matched metabolites
Phenylalanine, tyrosine and tryptophan biosynthesis	1/4	<0.001	tyrosine
Tyrosine metabolism	1/42	<0.001	tyrosine
Ubiquinone and other terpenoid-quinone biosynthesis	1/9	<0.001	tyrosine
Phenylalanine metabolism	1/10	<0.001	tyrosine
Tryptophan metabolism	1/41	<0.001	tryptophan
Cysteine and methionine metabolism	1/33	<0.001	methionine
Aminoacyl-tRNA biosynthesis	6/48	<0.001	histidine, methionine, valine, isoleucine, tryptophan, tyrosine
Histidine metabolism	1/16	<0.001	histidine
β-Alanine metabolism	1/21	<0.001	histidine
Pantothenate and CoA biosynthesis	1/19	0.001	valine
Valine, leucine and isoleucine degradation	2/40	0.001	isoleucine, valine
Valine, leucine and isoleucine biosynthesis	2/8	0.001	isoleucine, valine
Arachidonic acid metabolism	1/36	0.002	phosphatidylcholine
Linoleic acid metabolism	1/5	0.002	phosphatidylcholine
α-Linolenic acid metabolism	1/13	0.002	phosphatidylcholine
Sphingolipid metabolism	1/21	0.002	sphingomyelin
Glycerophospholipid metabolism	2/36	0.003	phosphatidylcholine, lysophosphatidylcholine

Supplementary Figure S1. The correlation between selected metabolites and clinical parameters. (A) Heatmap of the hierarchical clustering. The dendrogram on top shows the clustering of patients, and the dendrogram on the side shows the clustering of features. The colors on top of the heatmap represent patients at early or advanced stage disease. The colors in the heatmap represent normalized intensities, scaled to a mean of zero and unit variance for each feature. (B) Pathway enrichment of selected metabolites. Significantly enriched pathways from selected metabolites are illustrated in dot plots. aa: acyl-acyl; ae: acyl-alkyl; ADMA: asymmetric dimethylarginine; OH: hydroxy; lysoPC: lysophosphatidylcholine; PC: phosphatidylchololine; SM: sphinogomyelin.

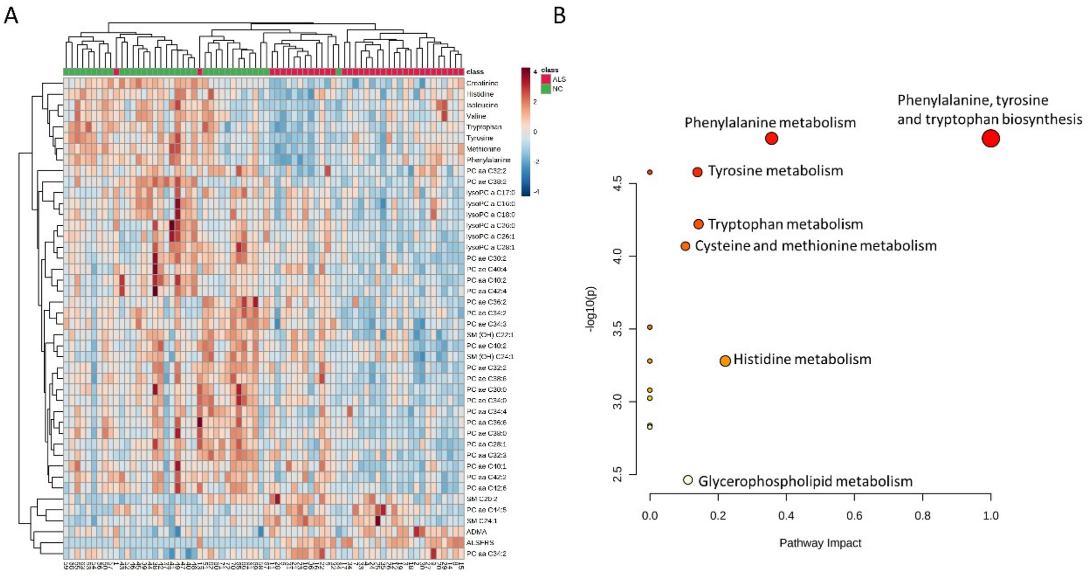

Supplement: Supplementary file 1 [file biomedicines-09-01944-s001.zip › biomedicines-1507453-supplementary.pdf]
